# Improving Fruit and Vegetable Consumption: Use of Farm-to-Consumer Venues Among US Adults

**Published:** 2011-02-15

**Authors:** Heidi Michels Blanck, Linda Nebeling, Amy L. Yaroch, Olivia M. Thompson

**Affiliations:** Division of Nutrition, Physical Activity, and Obesity, Centers for Disease Control and Prevention; National Cancer Institute, Rockville, Maryland; The Center for Human Nutrition, Omaha, Nebraska, and National Cancer Institute, Rockville, Maryland; The University of Nebraska Medical Center, College of Public Health, Department of Health Promotion, Social and Behavioral Health, Omaha, Nebraska

## Abstract

Improvements to the food environment including new store development and more farm-to-consumer approaches (ie, farmers' markets, roadside stands, pick-your-own produce farms, or community-supported agriculture programs) may aid Americans in making healthier dietary choices. We analyzed data from a subset of respondents (N = 1,994) in the National Cancer Institute's Food Attitudes and Behaviors Survey, a mail survey of US adults. We determined associations between primary grocery shoppers' region and sociodemographic characteristics and frequency of purchasing fruits and vegetables in the summer from farm-to-consumer venues. A little more than one-quarter (27%) of grocery shoppers reported a frequency of at least weekly use of farm-to-consumer approaches. Older adults and respondents who live in the Northeast were most likely to shop farm-to-consumer venues at least weekly, and no differences were found by sex, race/ethnicity, education, or annual household income. These findings suggest that farm-to-consumer venues are used by many Americans and could be expanded to increase access to fruits and vegetables.

## Objective

Eating a well-balanced diet that contains high amounts of fruits and vegetables helps prevent chronic diseases (1); however, few Americans consume recommended amounts (2). People from low-income, minority, and rural populations often have limited access to stores that sell healthful foods such as fruits and vegetables, and they have disproportionally higher prevalences of diet-related diseases (3,4). Improvements to the food environment may aid people in making more healthful dietary choices. Whereas establishing a supermarket can take many years, creation of farmers' markets and stands takes less time and can have a positive effect on a community's food supply and economy (3,5). There has been little assessment to determine the reach of farm-to-consumer venues (ie, farmers' markets, roadside stands, pick-your-own produce farms, or community-supported agriculture programs). We used a population-level mail survey of US adults to describe characteristics of shoppers by shopping venue and to assess associations between characteristics of shoppers (region and sociodemographics) and the summertime purchase of fruits and vegetables from farm-to-consumer venues.

## Methods

We analyzed cross-sectional data from the National Cancer Institute's Food Attitudes and Behaviors (FAB) Survey, a mail survey of US adults that was conducted from October through December of 2007. The study was approved by the National Cancer Institute's institutional review board.

Participants of the FAB Survey were recruited via household quota sampling through Synovate Global Opinion Panels (N = 450,000) (Synovate, Inc, Chicago, Illinois). A stratified random sampling of the panel was used to identify 5,803 potential respondents. The sample was balanced by region, annual household income, population density, age, and household size, and non-Hispanic blacks were oversampled (target, 25%). A total of 3,418 surveys were returned, 20 of which were incomplete, yielding a response rate of 59%. This sample was approximately 48% male and 28% non-Hispanic black, and most respondents had a high school degree (58%), an annual household income less than $50,000 (52%), and an age approximately evenly split between the categories of 18 to 34 years, 35 to 54 years, and 55 years or older.

Participants of the FAB Survey were asked "Who is the primary food shopper(s) in your household?" We obtained our sample (N = 1,994) by including only those respondents who identified themselves as their household's primary grocery shopper. Two follow-up questions were, "Where does the primary food shopper go grocery shopping?", and "In the summer, how often does the primary food shopper get fruits and vegetables from a farmers' market, roadside stand, pick-your-own produce farm, or community-supported agriculture program?"

Analyses were conducted by using SAS version 9.2 (SAS Institute, Inc, Cary, North Carolina). Data on sex, race/ethnicity, age, education, and annual household income were weighted based on the 2000 US Census to ensure that statistical estimates were more representative of the US household population; therefore, we report prevalence and standard errors. We used *χ* ^2^ tests to evaluate use of shopping venues, and significance was set at *P* ≤ .001 (2-sided α) to account for multiple testing. Odds ratios (ORs) and corresponding 95% confidence intervals (CIs) were computed for at least weekly summertime use (versus less often) by using multivariable logistic regression and controlling for sex, age, race/ethnicity, education, annual household income, and region.

## Results

Our sample of primary grocery shoppers differed somewhat from the overall FAB Survey sample. The sample of primary grocery shoppers was 72% female, 74% aged 35 years or older, 78% non-Hispanic white, and 12% non-Hispanic black (Table 1). Moreover, 42% had a high school degree or less, and most (63%) had an annual household income of less than $50,000. Most shoppers (86%) reported shopping at large-chain grocery stores or supermarkets, and 20% reported shopping at farmers' markets or cooperatives (Table 1). Variations in shopping venues by primary shopper characteristics were observed (Table 1). For example, use of large-chain stores did not vary by sex or race/ethnicity but did differ by age, education, household income, and region. Use of farmers' markets or cooperatives did not differ by sex, age, race/ethnicity, income, or region. However, use of farmers' markets or cooperatives increased by education level.

Of all 1,994 respondents, 1,852 (93%) responded when asked how often in the summer fruits and vegetables were purchased from farm-to-consumer venues, and 27% reported using these various venues at least weekly (Table 2). Compared with adults aged 18 to 34 years, adults aged 35 to 54 years and aged 55 years or older were more likely to purchase fruits and vegetables at least weekly during the summer from farm-to-consumer venues. Compared with adults who live in the West, adults who live in the Northeast were more likely to purchase fruits and vegetables at least weekly during the summer from farm-to-consumer venues. No associations were observed for sex, race/ethnicity, education, or annual household income (Table 2).

## Discussion

Our findings suggest that farm-to-consumer venues have the potential to reach many Americans and can augment supermarkets and grocery stores as places to obtain fruits and vegetables. Farmers' markets and other farm-to-consumer venues have increased throughout the United States in recent years. The US Department of Agriculture (USDA) has reported substantial growth in number of farmers' markets; there were 1,755 in 1994 and 5,274 in 2009 (6). Assessment of state-level policies also finds legislative support for farmers' markets; since 2001, 24 states have passed bills (7). Findings from evaluations of several USDA farmers' market programs suggest that program participation is associated with several benefits, including plans to consume more fruits and vegetables and, in most studies, improvement in fruit and vegetable consumption (8-10).

Our study has limitations. We used cross-sectional data, which limit inferences. The FAB survey response rate of 59%, although similar to response rates of other public health surveys that use random-digit–dial methods, was low; therefore, estimates may have been over- or underestimated. Although non-Hispanic blacks were oversampled as part of the study design, the sample size for other racial/ethnic groups was not sufficient. Finally, we cannot tell whether a no response meant that the respondent had a venue available but did not use it or that the venue was not available.

Many communities continue to focus on improving food environments through environmental and policy strategies to improve both the health and lives of their citizens through better nutrition (11,12) and the local economy through greater agricultural production of specialty crops such as fruits and vegetables. Therefore, expansion of farm-to-consumer venues should be considered for future public health initiatives. As the approaches to improving food environments increase, so will the need for evaluations that address their individual and community benefits.

## Figures and Tables

**Table 1:** Sociodemographic Characteristics of Primary Grocery Shoppers, by Grocery Shopping Location,^a^ Food Attitudes and Behaviors Survey, United States, 2007

**Characteristics**	No., %^b^	Large-Chain Stores, % (SE)	Natural Markets, % (SE)	Small Local Stores, % (SE)	Warehouse Club Stores, % (SE)	Discount Superstores, % (SE)	Farmers' Markets/Co-operatives, % (SE)
Total	1,994 (100)	86.4 (0.9)	10.3 (0.7)	21.3 (1.0)	30.2 (1.1)	52.2 (1.2)	20.0 (1.0)
**Sex**							
Male	414 (27.6)	88.1 (1.8)	11.2 (1.7)	21.5 (2.2)	28.3 (2.4)	43.5 (2.7)^c^	21.7 (2.3)
Female	1,536 (72.4)	85.7 (1.0)	10.1 (0.8)	21.2 (1.1)	31.0 (1.2)	55.7 (1.4)	19.5 (1.1)
**Age, y**							
18-34	475 (25.9)	82.6 (1.9)^c^	13.4 (1.7)	20.1 (2.0)	30.1 (2.2)	61.7 (2.4)^c^	18.5 (2.0)
35-54	814 (37.8)	85.6 (1.4)	8.0 (1.0)	21.6 (1.6)	31.6 (1.8)	53.1 (1.9)	18.6 (1.5)
≥55	665 (36.3)	89.6 (1.3)	10.7 (1.3)	21.2 (1.7)	28.7 (1.9)	45.1 (2.1)	22.6 (1.8)
**Race/ethnicity**							
Non-Hispanic white	1,290 (77.9)	86.2 (1.0)	10.1 (0.9)	20.7 (1.2)	28.5 (1.3)	50.1 (1.5)^c^	19.0 (1.1)
Non-Hispanic black	503 (12.4)	87.8 (1.6)	10.4 (1.4)	23.1 (2.0)	32.7 (2.2)	59.4 (2.3)	21.1 (1.9)
Other	157 (9.8)	85.8 (2.9)	12.9 (2.7)	24.1 (3.6)	38.8 (4.0)	55.9 (4.1)	26.0 (3.9)
**Education**							
<High school	213 (12.5)	82.4 (2.8)^c^	4.5 (1.5)^c^	23.8 (3.1)	21.3 (3.0)^c^	55.9 (3.7)^c^	13.2(2.4)^c^
High school diploma	565 (29.9)	82.9 (1.8)	4.4 (0.9)	24.8 (2.0)	25.1 (2.0)	58.2 (2.3)	18.5 (1.8)
Some college	605 (30.3)	89.0 (1.4)	12.3 (1.5)	19.0 (1.8)	31.8 (2.1)	52.9 (2.2)	21.2 (1.9)
≥College degree	564 (27.3)	88.9 (1.5)	17.4 (1.7)	18.4 (1.8)	37.9 (2.2)	43.6 (2.3)	23.3 (2.0)
**Income, $**							
<25,000	601 (34.1)	85.4 (1.6)^c^	6.9(1.2)^c^	24.2 (1.9)	19.4(1.8)^c^	59.5 (2.2)^c^	21.2 (1.9)
25,000-49,999	531 (28.8)	82.9 (1.8)	8.1 (1.3)	22.3 (2.0)	29.4 (2.2)	54.1 (2.4)	18.5 (1.8)
50,000-74,999	341 (15.3)	90.1 (1.7)	10.0 (1.7)	18.3 (2.2)	39.3 (2.8)	50.6 (2.9)	19.2 (2.3)
≥75,000	521 (21.8)	90.1 (1.4)	18.7 (1.7)	17.3 (1.7)	41.6 (2.2)	39.4 (2.2)	20.1 (1.8)
**Region**							
West	355 (19.1)	86.2 (2.0)^c^	20.0 (2.2)^c^	17.5 (2.2)^c^	46.1 (2.8)^c^	43.0 (2.9)^c^	19.0 (2.2)
Midwest	425 (22.3)	85.1 (1.9)	6.4 (1.6)	28.3 (2.4)	23.0 (2.1)	48.8 (2.6)	18.4 (2.1)
Northeast	386 (19.2)	92.2 (1.6)	10.3 (1.6)	23.7 (2.4)	33.4 (2.6)	42.8 (2.8)	26.2 (2.5)
South	828 (39.1)	84.5 (1.4)	7.9 (1.0)	17.9 (1.5)	24.7 (1.6)	63.3 (1.9)	18.3 (1.5)

Abbreviation: SE, standard error.

a Grocery shopping survey question response options: large-chain grocery stores or supermarkets, natural or organic supermarkets (such as Whole Foods Market), small local stores or corner stores, convenience stores (such as 7-Eleven or mini-market), warehouse club stores (such as Sam's Club or Costco), discount superstores (such as Walmart), online delivery (such as Peapod or Fresh Direct), ethnic markets, and farmers' markets/cooperatives. Less than 1% of the sample reported shopping at ethnic markets or using online delivery services (data not shown).

b The values for number of respondents are unweighted, and the percentages are weighted. Numbers may not sum to total because of missing data.

c *P* ≤ .001. *P* values obtained using *χ* ^2^ test.

**Table 2:** Primary Grocery Shoppers' Weekly Frequency of Obtaining Fruits and Vegetables From Farm-to-Consumer Venues^a^ During Summer Months, by Sociodemographic Characteristics, Food Attitudes and Behaviors Survey, United States, 2007

Characteristic	No. (%)^b^	At Least Once Per Week	
		Yes, % (SE)	OR (95% CI)^c^
Total	1,852 (100)	26.9 (1.12)	—
**Sex**			
Male	388 (27.5)	24.3 (2.4)	1 [Reference]
Female	1,464 (72.5)	27.9 (1.3)	1.22 (0.9-1.64)
**Age, y**			
18-34	456 (26.3)	20.0 (2.0)	1 [Reference]
35-54	775 (37.9)	27.1 (1.8)	1.47 (1.07-2.00)
≥55	621 (35.7)	31.8 (2.0)	1.91 (1.39-2.64)
**Race/ethnicity**			
Non-Hispanic white	1,240 (78.6)	27.4 (1.3)	1 [Reference]
Non-Hispanic black	467 (12.0)	23.8 (2.0)	0.92 (0.69-1.22)
Other	145 (9.4)	26.7 (3.9)	1.10 (0.70-1.64)
**Education**			
<High school	180 (11.0)	26.2 (3.6)	1 [Reference]
High school diploma	540 (30.1)	27.3 (3.6)	0.99 (0.64-1.53)
Some college	582 (30.9)	27.6 (2.0)	1.08 (0.70-1.67)
≥College degree	550 (27.9)	25.6 (2.0)	0.94 (0.60-1.49)
**Income, $**			
<25,000	517 (31.5)	26.8 (2.2)	1 [Reference]
25,000-49,999	500 (29.6)	24.9 (2.1)	0.96 (0.69-1.33)
50,000-74,999	333 (16.1)	26.8 (2.5)	1.10 (0.77-1.58)
≥75,000	502 (22.8)	29.8 (2.1)	1.19 (0.85-1.67)
**Region**			
West	328 (19.4)	24.5 (2.5)	1 [Reference]
Midwest	393 (22.0)	27.1 (2.4)	1.20 (0.83-1.72)
Northeast	362 (19.3)	34.3 (2.7)	1.62 (1.13-2.32)
South	769 (39.2)	24.4 (1.7)	1.10 (0.76-1.48)

Abbreviations: SE, standard error; OR, odds ratio; CI, confidence interval.

a Farm-to-consumer venues are farmers' markets, roadside stands, pick-your-own produce farms, or community-supported agriculture programs.

b The values for number of respondents are unweighted, and the percentages are weighted.

c Models are adjusted for sex, age, race/ethnicity, education, annual household income, and region.
